# JCI Accreditation and Outpatient Surgery: A Scoping Review of Evidence on Healthcare Quality, Risk Management, and Policy

**DOI:** 10.3390/healthcare14172858

**Published:** 2026-09-04

**Authors:** Kaisar Kudabayev, Aigul Ismailova, Kenesh Dzhusupov, Bakhyt Yeleussizova, Gulmira Zhauarova, Oxana Tsigengagel

**Affiliations:** 1 Department of Epidemiology and Biostatistics, Astana Medical University, Astana 010000, Kazakhstan; 2 Department of Public Health, International Higher School of Medicine, Bishkek 720054, Kyrgyzstan; k.dzhusupov@ism.edu.kg; 3 International Medical Faculty, Osh State University, Osh 723500, Kyrgyzstan; 4 Department of Management and Quality, Turkestan City Polyclinic, Turkestan 161200, Kazakhstan; 5 Department of Anesthesiology, National Research Cardiac Surgery Center, Corporate Fund “University Medical Center”, Astana 010000, Kazakhstan

**Keywords:** Joint Commission International, JCI accreditation, outpatient surgery, healthcare quality, risk management, scoping review

## Abstract

**Background**: The global healthcare landscape is being increasingly shaped by two pivotal trends: the adoption of international quality frameworks, such as Joint Commission International (JCI) accreditation, and the strategic shift towards resource-efficient models like outpatient surgery. While both areas have been extensively addressed in the healthcare literature, empirical evidence concerning their intersection remains limited. This scoping review aimed to systematically map the available literature, identify critical evidence gaps at the intersection of JCI accreditation and outpatient surgery, and examine implications for healthcare risk management and policy development. **Methods**: The scoping review was performed in accordance with the PRISMA-ScR guidelines. A dual-track evidence-mapping strategy was implemented across seven electronic databases and platforms (PubMed/MEDLINE, Scopus, Web of Science, Cochrane Library, eLIBRARY.ru, CyberLeninka, and Google Scholar) to search for publications published between January 2000 and May 2026. The final database searches were conducted on 15 May 2026. Data extraction was centered on study design, interventions, and key findings related to quality and risk management. **Results**: A total of 80 publications were included. The evidence comprised two complementary streams: literature concerning JCI accreditation and healthcare quality and safety, predominantly in hospital and general healthcare settings, and literature concerning outpatient, ambulatory, and day-surgery quality, safety, and hospital-substituting technologies. No empirical study directly evaluating the implementation or impact of JCI accreditation within outpatient surgery centers was identified. **Conclusions**: This scoping review identified a critical empirical evidence gap concerning the integration of accreditation and quality and risk-management frameworks in outpatient surgical settings. A hypothesis-generating conceptual framework is proposed to guide future empirical research evaluating whether and how JCI accreditation influences quality, safety, organizational, and economic outcomes in outpatient surgical settings.

## 1. Introduction

The global pursuit of high-quality, safe, and efficient healthcare has propelled two transformative forces to the forefront of health system reform. The first is the rise of international accreditation, with Joint Commission International (JCI) representing a prominent international framework for institutionalizing patient safety and quality management [1,2,3,4,5]. The second is the strategic shift toward resource-optimizing delivery models, exemplified by the rapid global expansion of outpatient surgery, which now accounts for a majority of elective procedures in many developed nations [6,7,8,9,10]. These parallel movements represent the twin pillars of modern healthcare delivery: one focused on quality assurance, the other on operational efficiency.

Despite the parallel rise of these two powerful trends, a critical disconnect exists in both research and practice. JCI standards were historically developed and validated within complex hospital environments [4,11,12]. Conversely, outpatient surgery centers (OSCs) operate under a fundamentally different model characterized by high patient turnover, limited post-operative observation, and distinct logistical risks. This misalignment creates a critical blind spot for policymakers and healthcare leaders: Are we effectively applying hospital-centric quality standards to ambulatory settings, or are we failing to address unique risks? The analytical link between international accreditation standards and the specific operational risks of outpatient surgery remains a critically under-explored area in the current evidence base.

To address this critical gap, a scoping review was determined to be the most appropriate methodology. This approach is ideal for mapping the extent, range, and nature of evidence in a broad and heterogeneous field, identifying knowledge gaps, and summarizing findings without the requirement for formal quality appraisal, which is needed in a systematic review [13,14]. The present scoping review aims to systematically map two complementary bodies of evidence concerning JCI accreditation and outpatient surgery and to determine whether empirical evidence exists at their intersection. Specifically, this review addressed the following research questions:

What is the extent and nature of the existing literature at the intersection of JCI accreditation and outpatient surgery?

What are the key thematic areas and conceptual frameworks present in the current evidence, particularly concerning risk management, quality, and policy?

What are the critical evidence gaps regarding the implementation and impact of JCI standards in outpatient surgical settings that require future empirical investigation?

The present scoping review was structured using the Population, Concept, and Context (PCC) framework. The Population included healthcare organizations and professionals involved in accreditation and quality improvement, as well as patients and populations receiving ambulatory surgical care. The Concept encompassed JCI accreditation and quality and safety standards, as well as clinical quality indicators and risk-management mechanisms relevant to outpatient surgery. The Context included hospital and general healthcare settings in which accreditation-related evidence was generated and hospital-based or freestanding outpatient, ambulatory, and day-surgery settings in which evidence concerning surgical quality and risk management was identified.

## 2. Materials and Methods

### 2.1. Study Design

This study was designed as a scoping review to systematically map the existing literature at the intersection of international accreditation and outpatient surgery and to identify directions for future research. The methodology was guided by the foundational framework proposed by Arksey and O’Malley [13], and this report adheres to the Preferred Reporting Items for Systematic Reviews and Meta-Analyses extension for Scoping Reviews (PRISMA-ScR) guidelines [14]. A scoping review approach was selected as the ideal methodology for mapping a broad and heterogeneous body of evidence where a comprehensive systematic review would be premature.

### 2.2. Information Sources and Search Strategy

A comprehensive literature search was conducted across seven electronic databases and platforms: PubMed/MEDLINE, Scopus, Web of Science, the Cochrane Library, eLIBRARY.ru (Russian Science Citation Index), CyberLeninka, and Google Scholar. The literature search covered the period from January 2000 to May 2026, with the final database searches conducted on 15 May 2026. The complete database-specific search strategies and Boolean search strings are provided in Supplementary Table S1.

The search strategy combined MeSH terms and free-text keywords using Boolean operators (AND, OR) across three primary domains: (1) international accreditation, (2) surgical settings, and (3) quality and risk outcomes. An example of the search string used in PubMed is: (“Joint Commission International” OR “JCI”) AND (“ambulatory surgery” OR “outpatient surgery”) AND (“patient safety” OR “risk management” OR “healthcare quality”).

### 2.3. Eligibility Criteria

To address the evidence gap without presupposing the existence of studies directly examining the intersection of JCI accreditation and outpatient surgery, we adopted a dual-track evidence-mapping strategy. Track 1 focused on evidence concerning JCI accreditation, healthcare quality and safety standards, and risk management in hospital and general healthcare settings. Track 2 focused on evidence concerning outpatient, ambulatory, and day-surgery quality, safety, clinical pathways, risk management, and hospital-substituting technologies. The two evidence streams were subsequently mapped against each other to determine whether direct empirical evidence existed at their intersection.

Eligible publication types included original research (quantitative and qualitative observational studies), systematic reviews, implementation reports, analytical reviews, and official policy documents and standards from international and national quality organizations, such as the World Health Organization (WHO) [15], the International Society for Quality in Health Care (ISQua) [16], and Joint Commission International (JCI) [4], Accreditation Canada [17], ACHS International [18], and the American Accreditation Commission International (AACI) [19]. Publications addressing neither JCI/accreditation-related quality and safety frameworks nor outpatient/ambulatory surgery-related quality, safety, or risk-management issues were excluded (Table 1). In addition, conference abstracts without full-text versions, unverified editorials, and duplicate publications were excluded.

### 2.4. Study Selection

The study selection process followed a systematic, two-stage approach conducted by two independent reviewers. In the first stage, titles and abstracts were screened for relevance against the predefined eligibility criteria. In the second stage, full-text articles of potentially eligible records were retrieved and assessed. Any disagreements between reviewers at either stage were resolved through discussion or consultation with a third senior reviewer. The overall study selection process and final inclusion of 80 sources are detailed in the PRISMA flow diagram presented in Section 3.1.

### 2.5. Data Charting and Synthesis

A standardized data extraction form was developed and used by two independent researchers to chart relevant information from the included studies. The extracted data included publication details (year, country), study design, population characteristics, and primary findings related to the review’s objectives.

A descriptive-thematic synthesis approach was employed to analyze the charted data. First, the characteristics of the included literature were summarized using descriptive statistics (frequency, percentage). Second, the findings were systematically coded and organized into major thematic domains, focusing on their relevance to risk management strategies and global healthcare policy. This synthesis explicitly aimed to map critical evidence gaps, particularly the lack of empirical data on the implementation and impact of JCI accreditation within outpatient surgical settings, thereby providing a foundational framework for future research.

## 3. Results

### 3.1. Study Selection and Characteristics of Included Publications

**Figure 1:**
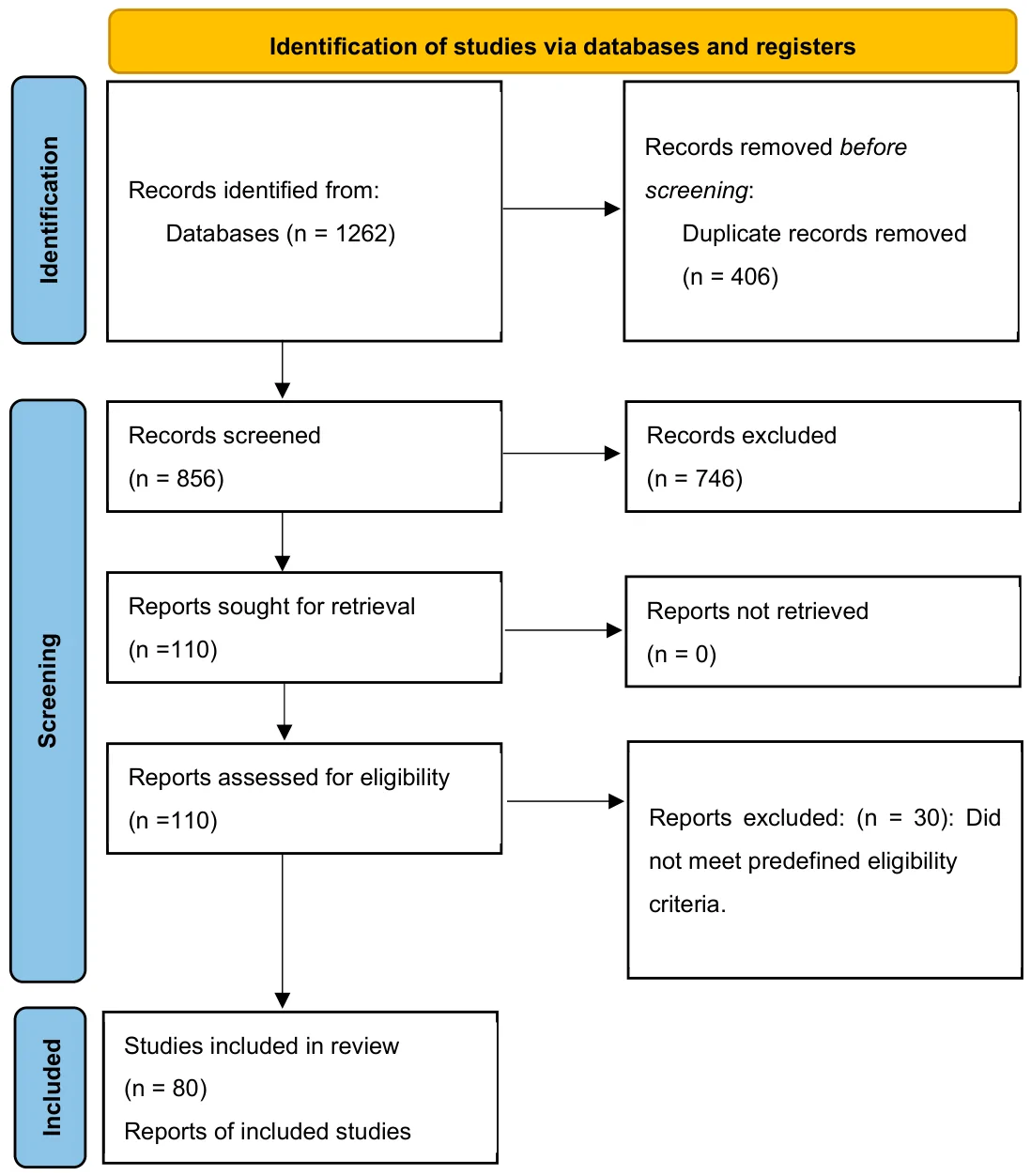
PRISMA flow diagram of the study selection process.

The literature search and screening process are summarized in the PRISMA flow diagram in Figure 1 [14]. The initial search across the seven electronic databases and platforms yielded 1262 records. After the removal of 406 duplicates, 856 records were screened by title and abstract, leading to the retrieval and full-text assessment of 110 articles. Of these, 30 reports were excluded because they did not meet the predefined eligibility criteria. Ultimately, 80 publications met all eligibility criteria and were included in the scoping review.

The 80 included sources were subsequently classified according to their primary evidence domain. The classification distinguished evidence concerning JCI accreditation and healthcare quality and safety from evidence concerning outpatient, ambulatory, and day-surgery care. Sources were additionally examined for direct empirical evidence addressing the intersection of JCI accreditation and outpatient surgery. The complete source-level classification is provided in Supplementary Table S2.

The included literature is methodologically diverse and spans a wide range of geographical contexts. Table 2 provides a summary of the characteristics of 15 selected publications to illustrate the breadth of the evidence base.

### 3.2. Overview of Thematic Areas

An analysis of the 80 included publications revealed a methodologically diverse evidence base, as detailed in Table 3. The most common publication types were original observational research (n = 29, 36.25%) and reports describing implementation experience (n = 18, 22.50%). This composition indicates a field characterized by both empirical investigation and practical application reports relevant to policy and risk assessment.

A thematic analysis showed that the literature is concentrated in several key areas (Table 4). The most prevalent themes concerning accreditation were “The Impact of Accreditation on Clinical Outcomes” (18 mentions) and “Impact on organizational processes and security” (15 mentions), reflecting the extensive focus of existing research on the effects of accreditation within hospital settings.

An equally prominent thematic cluster, comprising 15 publications, focused on “Hospital-substituting technologies in surgery” (Table 4). These publications examined the feasibility and safety considerations associated with shifting selected surgical procedures from inpatient to ambulatory settings, including patient selection, clinical pathways, and operational requirements. This body of evidence highlights the importance of standardized processes and appropriate risk-management mechanisms when expanding outpatient surgical care.

### 3.3. Thematic Findings on JCI Accreditation

The review identified a substantial and mature body of evidence focused on the implementation and effects of JCI accreditation. The key thematic findings are synthesized below.

#### 3.3.1. Association with Clinical Outcomes and Organizational Safety

A recurrent theme in the literature is the association between JCI accreditation and improvements in clinical outcomes and organizational safety [20,21,22,33,34,35]. Studies from various national contexts, including the United States, South Korea, and Taiwan, report associations with reduced mortality and readmission rates [20,21,34]. Furthermore, the evidence indicates that accreditation is a catalyst for organizational change, promoting the standardization of processes and fostering a culture of safety [25,26,36,37,38,39,40].

#### 3.3.2. Impact on Patients and Medical Staff

The literature reports a multifaceted impact on key stakeholders. For patients, the association is predominantly positive, with studies linking accreditation to increased patient support and satisfaction [36,38,40,41,42]. For medical staff, a dual impact is noted: multiple sources identify increased stress and workload during the preparatory phases [24,43,44,45], while others document long-term benefits such as improved safety culture and enhanced professional satisfaction post-accreditation [23,46,47,48,49,50,51].

#### 3.3.3. Economic Impact and Application in Specialized Contexts

The economic effects of accreditation are reported as highly context-dependent [22,27,35,47]. While some studies identify specific cost savings [52], the overall evidence on direct financial benefits is inconsistent. The literature does, however, demonstrate the versatility of JCI standards, with numerous reports of successful adaptation to specialized clinical areas, such as pediatric oncology [28], orthopedic surgery [53], oncology [54], and medication management [55,56].

### 3.4. Thematic Findings on Outpatient Surgery

Parallel to the literature on accreditation, the review mapped an extensive evidence base on the efficacy of outpatient surgery [6,7,8,9,30,57,58,59,60,61,62,63]. This body of work describes potential medical, social, and economic advantages associated with shifting selected surgical procedures to ambulatory settings, including reduced treatment times, lower complication rates, and potential cost savings compared with inpatient care [6,7,8,9,30,59,64].

### 3.5. The Identified Evidence Gap at the Intersection of JCI and Outpatient Surgery

The primary finding of this scoping review is the identification of a critical evidence gap in the scientific literature. Despite the extensive bodies of research on JCI accreditation in hospital settings and the expanding literature on outpatient surgical care, the systematic mapping identified no empirical studies directly examining the implementation or impact of JCI accreditation within outpatient surgery centers.

Specifically, no publications were identified that directly evaluated the adaptation of JCI standards within outpatient surgery centers, nor were studies found that measured the associations of such accreditation with clinical, organizational, or economic outcomes in an ambulatory surgical context. The analysis indicates that these two fields, while central to modern healthcare delivery, currently function as separate evidence streams within the published scientific literature. This constitutes a significant empirical gap for healthcare policy, risk management, and clinical practice.

## 4. Discussion

### 4.1. Summary of Principal Findings from the Scoping Review for Risk Management and Healthcare Policy

This scoping review systematically mapped the existing literature on Joint Commission International (JCI) accreditation and the expansion of outpatient surgical care, focusing on their roles in risk management and their healthcare policy implications. Three principal findings emerged from this mapping exercise.

First, the mapped literature predominantly focuses on JCI accreditation in hospital settings. It consistently describes associations with improved clinical outcomes, organizational processes, and safety culture across diverse settings, though the magnitude of association varies considerably. Studies from South Korea [21], the United States [20], and Taiwan [34] reported lower mortality in accredited hospitals, while research from Italy [65] found no significant differences, suggesting that the effectiveness of accreditation as a risk management tool can vary by context and baseline quality levels [34,35].

Second, the mapped evidence on outpatient surgical care demonstrates substantial potential for medical, social, and economic efficiency, with up to 55–80% of procedures performed ambulantly in developed health systems [8,9]. However, these outcomes are consistently linked to organizational conditions: appropriate patient selection, qualified staffing, adequate facilities, and clear protocols. The efficient delivery of care through outpatient surgery represents a key policy lever for resource optimization and improved patient access.

Third, the mapped experience of CIS countries, particularly Kazakhstan, illustrates how international standards can be adapted to national contexts [2,66]. Kazakhstan’s development of an ISQua-accredited national accreditation system [16,56,57,67,68,69,70,71], alongside JCI-accredited institutions [3,68], offers a potential model for emerging economies. Local studies from Kazakhstan further enrich this context by highlighting regional challenges, such as the epidemiology of offences against health [32], patient satisfaction and disparities related to health insurance status [72], and healthcare professionals’ perspectives on systemic care challenges [73].

A critical and consistent gap identified across the mapped literature is the lack of empirical studies directly examining Joint Commission International (JCI) accreditation within outpatient surgery centers, representing an unmapped area for integrated risk management and policy evaluation.

### 4.2. Mechanisms: How Might Accreditation Produce Effects for Risk Management?

Understanding the mechanisms through which accreditation influences outcomes is essential for interpreting the mapped evidence and guiding risk management implementation. Four interconnected mechanisms emerge from the literature: standardization of clinical processes, behavioral change through audit and feedback, resource allocation prioritization, and reputational motivation. These mechanisms help explain why accreditation associations vary, depending on baseline processes, resource availability, and local market conditions.

Standardization of clinical processes. JCI standards require explicit protocols for key clinical activities: surgical safety checklists, medication reconciliation, hand hygiene, and patient identification. Japanese studies on operative time suggest that standardization need not reduce efficiency and may, in fact, enhance it [29,74]. By reducing unwarranted variation, standardization minimizes errors and creates predictable workflows, thereby supporting operational risk reduction.

Behavioral change through audit and feedback. The accreditation process involves external surveyors observing practice, reviewing records, and providing feedback. This creates what might be termed a “visible accountability effect”: knowing that performance will be assessed against explicit standards modifies behavior. The hand hygiene improvements documented in ICU settings illustrate that this mechanism fosters sustained preparation that changes daily routines, embedding safety practices [75].

Resource allocation prioritization. Preparing for accreditation forces organizations to allocate resources toward quality-related infrastructure: staff training, equipment upgrades, and information systems. The medication management improvements reported in China [52] and Kazakhstan [56] required dedicated investment in formularies, monitoring systems, and personnel development. Accreditation thus functions as a mechanism for signaling organizational commitment to quality, both internally and externally, and prioritizes investment in risk mitigation.

Reputational motivation. JCI accreditation confers prestige and market differentiation. JCI accreditation was identified as a key factor in enhanced reputation and staff pride in qualitative assessments of hospital leadership [23,47]. This reputational effect may drive organizational commitment beyond what regulatory compliance alone would achieve, indirectly supporting sustained risk management efforts.

### 4.3. Why Might Findings Differ Across Settings and What Are the Implications for Policy?

The heterogeneity in study findings, as mapped in this review, is not merely methodological noise but reflects meaningful contextual variation that directly impacts healthcare policy and risk management strategies. Three contextual factors appear particularly important.

Baseline quality levels. The “ceiling effect” hypothesis that accreditation yields greater improvements where baseline quality is lower is supported by multiple observations. The dramatic mortality reductions reported in the Russian hospital [1] occurred in a setting starting from relatively low baseline compliance. Conversely, the Italian study that found no mortality differences was conducted in a health system with uniformly high baseline standards [65]. The systematic review by Vuohijoki et al. (2025) explicitly concluded that accreditation effects are more pronounced in developing countries, precisely where baseline quality is more variable [76]. These differences highlight the importance of policy decisions being tailored to the baseline risk profile of a healthcare system.

Regulatory environment. In countries with strong governmental regulation, accreditation may add less marginal value. A comparative study of different accrediting agencies in the United States found relatively small differences, suggesting that when minimum standards are already high, accreditation becomes more about differentiation than fundamental improvement [33]. In contrast, in settings where governmental oversight is weaker, accreditation may serve as a primary driver of quality, effectively serving as a de facto regulatory mechanism for risk control [11].

The mixed findings on staff perceptions, ranging from stress and burnout [45,47] to positive appreciation [43,44], likely reflect differences in how accreditation is implemented. Organizations that invest in preparation, communication, and support may realize the benefits while mitigating the burdens. The temporal pattern documented by Rhoden et al. (2021) shows that anxiety during preparation decreases after accreditation, suggesting that stress may be transient if the process is well-managed [24]. Furthermore, the baseline safety culture, patient safety culture indicators such as the intention to disclose medical errors [72], and determinants of patient satisfaction [77] can significantly influence the implementation success of accreditation efforts. The Palestinian studies on patient satisfaction illustrate another dimension of context: patient perceptions may be influenced by factors unrelated to accreditation (facility age, location, staffing levels) that confound simple comparisons [40,42]. This underscores the need for multi-site, controlled studies that account for contextual variables when assessing policy impacts.

### 4.4. The Synergy Argument: JCI Accreditation and Outpatient Surgery as an Integrated Conceptual Model

The central conceptual proposition emerging from this review is that the potential integration of Joint Commission International (JCI) accreditation principles with outpatient surgical care warrants empirical investigation. A critical observation emerging from the scoping review is that no empirical studies directly examining JCI-accredited outpatient surgery centers (OSCs) were identified. While the literature includes evidence concerning JCI accreditation in hospital settings [20,21,22,33,34,35] and separate evidence concerning outpatient surgical care [6,7,8,9,78], the combined implementation and effects of JCI standards in ambulatory surgical facilities remain insufficiently evaluated.

Crucially, in accordance with scoping review methodology [13,14], the absence of direct empirical evidence reflects an important knowledge gap. Accordingly, this section presents a hypothesis-generating conceptual framework rather than an empirically established model, intended to guide future empirical investigations and to clearly distinguish our conceptual synthesis from evidence-based findings.

**Figure 2:**
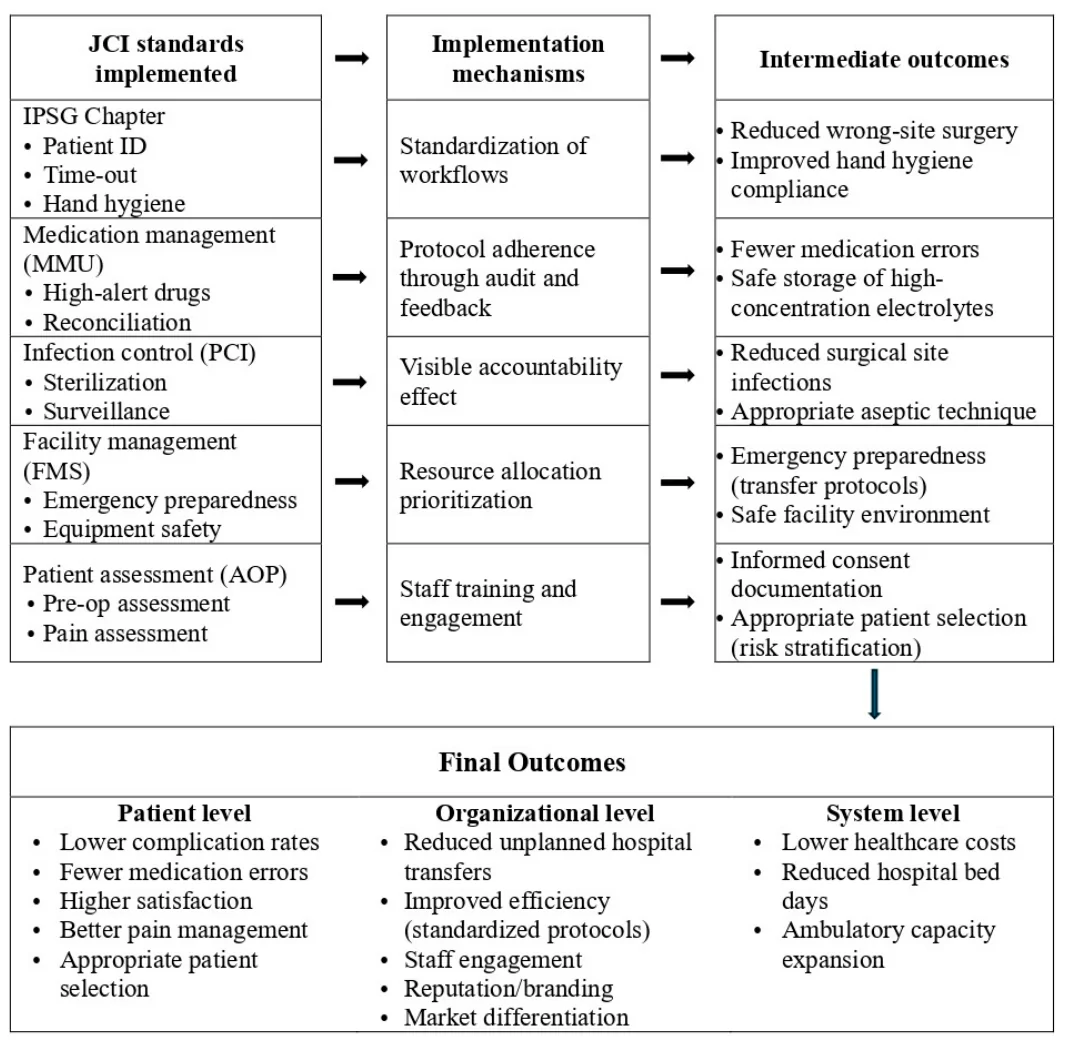
Proposed hypothesis-generating conceptual framework for the potential integration of JCI standards in outpatient surgery centers (OSCs).

Based on the synthesis of JCI standards [4,11] and the mechanisms of accreditation effects identified in Section 4.2, we propose a conceptual framework (Figure 2) for how JCI accreditation could potentially interface with outpatient surgical quality and safety.

The proposed framework hypothesizes that JCI standards could address specific operational risks in outpatient surgery, including higher patient turnover, shorter observation periods, and limited on-site resources for managing complications through the following.

International Patient Safety Goals (IPSGs): Correct patient identification, effective clinical communication, and surgical safety checklists may help address procedure-related risks when patients move rapidly through the system [4,11].

Medication Management and Use (MMU): Standardized medication reconciliation and safety protocols could reduce medication-related errors [52], which is particularly important when patients self-administer medications post-discharge with limited professional supervision.

Prevention and Control of Infections (PCI): Stringent sterilization and infection-surveillance protocols are important components of surgical safety. Continuous infection surveillance is particularly relevant because surgical site infections remain an important source of postoperative morbidity across surgical settings [79]. These considerations support the inclusion of ongoing infection surveillance among relevant quality and safety indicators for outpatient surgery centers.

Facility Management and Safety (FMS): Autonomous emergency preparedness and equipment safety protocols help ensure that physically separate OSCs maintain appropriate emergency preparedness and address environmental risks.

Assessment of Patients (AOP): Rigorous pre-operative risk stratification and standardized patient assessments minimize the likelihood of acute postoperative complications.

Conversely, OSCs may be well-suited to demonstrating JCI compliance. Studies on high-volume ambulatory procedures, including cataract surgery, suggest that standardization and protocol adherence may improve procedural efficiency [29,74]. However, whether such benefits can be specifically attributed to JCI accreditation in outpatient surgery remains to be empirically established. This suggests that ambulatory settings with focused, high-volume procedures may achieve efficiency gains from protocol adherence that are less visible in complex hospital environments.

Based on the evidence reviewed, we propose that the successful integration of JCI standards into outpatient surgical care requires attention to four domains: patient selection protocols (minimizing unplanned admissions), perioperative process standardization (streamlined for ambulatory workflows), discharge and follow-up systems (ensuring continuity of care), and emergency preparedness (protocols for transferring patients to acute care).

This integrated conceptual framework requires empirical testing. Priority research questions for risk management and healthcare policy include: Do JCI-accredited OSCs have lower complication rates than non-accredited OSCs? Does accreditation affect patient selection decisions? What are the cost implications of accreditation for OSCs? Evaluating this integration requires multi-dimensional measurement strategies. Drawing on established Donabedian structure-process-outcome frameworks [34,80], we propose the measurement approach previously presented in Table S2, complemented by an evaluation battery for JCI-accredited OSCs (Table 5).

The following hypotheses are proposed solely as hypothesis-generating propositions for future empirical testing and should not be interpreted as findings established by the present scoping review:

**H1.**  *JCI-accredited OSCs will have lower rates of wrong-site surgery, medication errors, and surgical site infections compared to non-accredited OSCs*.

**H2.**  *Compliance with International Patient Safety Goals will be higher in JCI-accredited OSCs and will correlate positively with patient satisfaction scores*.

**H3.**  *JCI-accredited OSCs will demonstrate more complete documentation of preoperative risk assessments and informed consent*.

**H4.**  *The standardization effect of JCI accreditation will be associated with reduced procedure times in high-volume ambulatory procedures*.

**H5.**  *Unplanned hospital transfer rates will be lower in JCI-accredited OSCs due to robust emergency preparedness and transfer protocols*.

#### Evaluation and Implementation

The implementation of JCI’s Outpatient Care Program involves approximately 600 evaluation items adapted to ambulatory workflows [46,72]. To enable rigorous testing of the integration synergy, we propose the evaluation battery for JCI-accredited OSCs presented in Table 5.

This measurement framework is intended to generate evidence to guide policy regarding Joint Commission International (JCI) accreditation in ambulatory settings, addressing the lack of direct empirical data identified in this review.

### 4.5. Implications for Policy and Practice

The findings mapped in this scoping review offer several critical implications for global healthcare policy and the strategic management of clinical risks. For healthcare systems evaluating the adoption of Joint Commission International (JCI) accreditation, the identified literature suggests that such frameworks may provide the most significant utility in contexts characterized by variable baseline quality and limited governmental oversight. In emerging economies, the pursuit of JCI accreditation is described as both a catalyst for organizational improvement and a mechanism for established international credibility; however, the substantial financial and structural investments required necessitate a cautious, often phased implementation strategy. The development of national accreditation systems that are incrementally aligned with international standards, as demonstrated in the Republic of Kazakhstan, provides a foundational model for other emerging healthcare systems seeking to balance global benchmarks with local scalability [31].

With respect to the expansion of outpatient surgical models, the evidence highlights a significant opportunity for resource optimization, with high-volume procedures suitable for ambulatory settings reportedly reaching 80% in mature healthcare systems [8,9]. Nevertheless, the literature underscores that these efficiency gains are strictly contingent upon the presence of rigorous organizational conditions and risk management protocols. Consequently, there is a clear requirement for policymakers to develop dedicated regulatory frameworks for outpatient surgery centers (OSCs) that specifically address facility requirements, specialized staff qualifications, standardized patient selection criteria, and continuous quality monitoring [15,60,70].

Furthermore, the evidence gap concerning the direct intersection of accreditation and ambulatory care presents an opportunity for future policy research. For jurisdictions planning to expand their ambulatory surgical capacity, the potential resource implications and feasibility of integrating JCI standards during the initial development of outpatient surgical services should be evaluated empirically rather than assumed to be superior to sequential implementation. Such evaluation should consider implementation costs, infrastructure readiness, workforce requirements, technical support, and governance capacity.

From a health policy perspective, implementation of complex quality and safety interventions in resource-constrained environments may depend on infrastructure readiness, appropriately trained personnel, technical support, sustainable financing, and effective governance [81].

### 4.6. Limitations

This scoping review has several limitations. First, consistent with the scoping review framework, a formal appraisal of the methodological quality of the included studies was not performed; therefore, this review maps the evidence rather than confirms its effectiveness. Because much of the empirical evidence included in the review is observational, causal inferences regarding the effects of accreditation cannot be established. The reported associations may also be influenced by residual confounding, selection bias, differences in baseline quality, and publication bias. Second, the heterogeneity of the literature precluded any form of meta-analysis. Third, the search was restricted to English and Russian language publications, which may have introduced a language bias. Finally, and most importantly, the central finding of this review—the absence of direct empirical evidence—means that the proposed integrated model (Figure 2) is conceptual and hypothesis-generating, requiring future empirical validation.

### 4.7. Future Research

The preceding analysis identifies several priority areas for future research that are crucial for advancing risk management and informing healthcare policy:

Rather than asking simply whether accreditation works, future research should examine how it functions across different contexts to achieve risk reduction. Mixed-methods studies combining quantitative outcomes with qualitative explorations of implementation processes would illuminate the mechanisms proposed in Section 4.2. Specifically, investigating how accreditation influences patient safety culture indicators, including the intention to disclose medical errors among health professionals [26,72], is essential.

Is accreditation more impactful in lower-baseline settings? How do organizational resources dictate implementation success? Research should investigate how national public health challenges (e.g., the epidemiology of offences against health) [32] and the determinants of patient satisfaction [77] interact with accreditation efforts to better inform context-specific policy decisions.

Prospective studies evaluating Joint Commission International (JCI) accredited outpatient surgery centers are urgently needed. These studies should compare clinical outcomes, economic costs, and patient experiences with non-accredited OSCs and traditional hospital-based care to empirically validate the proposed integrated risk management model.

Following organizations through multiple accreditation cycles would reveal whether risk reduction effects are sustained, amplified, or diminished over time. Comprehensive cost-effectiveness analyses encompassing both direct implementation costs and indirect benefits are required to inform long-term healthcare expenditure policy.

Research on adapting JCI standards to resource-constrained settings without compromising core safety principles will support wider global adoption. The Guatemalan pediatric oncology experience [28] and Kazakhstan’s development of an ISQua-recognized national accreditation system [16,31,68,82] offer foundational models for investigating how international standards can be effectively balanced with local implementation challenges.

## 5. Conclusions

This scoping review systematically mapped the evidence on JCI accreditation and outpatient surgery, identifying two complementary bodies of literature. While one predominantly addresses accreditation and quality in hospital and general healthcare settings and the other addresses quality, safety, and risk management in outpatient surgical care, no empirical studies directly evaluating the implementation or impact of JCI accreditation within outpatient surgery centers were identified.

To address this evidence gap, the review provides a hypothesis-generating conceptual framework and priority research questions to guide future empirical investigation. The proposed framework should be considered a conceptual research agenda rather than an evidence-based model of effectiveness. Closing this evidence gap is essential for developing evidence-informed policies to support patient safety and structured risk management in outpatient surgical settings.

## Figures and Tables

**Table 1:** Eligibility Criteria based on the Population-Concept-Context (PCC) Framework.

Component	Inclusion Criteria	Exclusion Criteria
Population	Medical organizations, surgical patients, healthcare professionals.	Studies not focused on healthcare settings or patient populations.
Concept	Implementation, impact, or perception of JCI standards; outpatient, ambulatory, or day surgery models.	Publications addressing neither JCI/accreditation-related quality and safety frameworks nor outpatient/ambulatory surgery-related quality, safety, or risk-management issues.
Context	Global healthcare settings; focus on quality, risk management, patient safety, or healthcare policy.	Studies with no relevance to quality, risk, or policy outcomes.
Study Types	Original research (quantitative, qualitative), systematic reviews, implementation reports, official documents.	Conference abstracts, editorials without original data, duplicate publications.
Language	Publications in English or Russian.	All other languages.

**Table 2:** Characteristics of Selected Included Publications (*N* = 15).

Author (Year) [Ref.]	Country/Region	Study Design	Key Focus Area	Main Finding Relevant to This Review
Impact on Clinical Outcomes				
Lam et al. (2018) [20]	USA	Multi-Hospital Observational Study	Accreditation and patient outcomes	Hospital accreditation was associated with lower 30-day mortality and readmission rates across US hospitals.
Chun et al. (2020) [21]	South Korea	Pre-Post Accreditation Comparison	Accreditation and in-hospital mortality	Accreditation was associated with reduced in-hospital mortality for patients with major cardiovascular diseases.
Alhawajreh et al. (2023) [22]	Global	Systematic Review	Impact of accreditation on quality	Confirmed a predominantly positive, though context-dependent, impact of accreditation on quality improvement indicators.
Impact on Stakeholders (Staff & Patients)				
Zhang et al. (2023) [23]	China	Qualitative Study	Hospital leaders’ perception of JCI	Identified enhanced reputation, branding, and staff pride as key motivations for pursuing accreditation.
Rhoden et al. (2021) [24]	Brazil	Longitudinal Study	Impact of accreditation on nursing staff	A temporary increase in anxiety and musculoskeletal pain was observed among nurses leading up to the accreditation audit.
Al-Awa et al. (2012) [25]	Saudi Arabia	Benchmarking Study	Patient safety culture	JCI-accredited hospitals demonstrated a significantly higher patient safety culture score compared to non-accredited hospitals.
Greenfield et al. (2019) [26]	Australia	Multi-Site Study (311 hospitals)	Accreditation and clinical care change	Found that accreditation stimulates change by enhancing patient engagement and providing greater support.
Economic & Specialized Applications				
Sriratanaban et al. (2020) [27]	Thailand	Cost–Benefit Analysis	Economic aspects of accreditation	Noted high implementation costs and the need for significant organizational restructuring, with mixed financial outcomes.
Day et al. (2013) [28]	Guatemala	Implementation Report	JCI in resource-limited settings	Demonstrated that JCI standards can be successfully adapted to improve pediatric oncology care even in resource-constrained settings.
Okumura et al. (2019) [29]	Japan	Observational Study	Standardization and efficiency	Standardization according to JCI accreditation was associated with significantly shortened cataract surgery times.
Outpatient Surgery Context				
Yarikov et al. (2024) [9]	Russia	Narrative Review	Capabilities of outpatient surgery	Outlines the modern capabilities and growing efficiency of outpatient surgery centers as an alternative to hospitalization.
Fleisher L.A. (2023) [30]	USA	Economic Review	Economics of ambulatory surgery	Analyzes the significant economic implications and cost-saving potential of shifting procedures to ambulatory settings.
Policy & Regional Adaptation (CIS)				
Khairullin et al. (2021) [3]	CIS/Global	Review	International accreditation in CIS	Provides an overview of JCI implementation in CIS clinics, contextualizing it within global practice.
Kaupbaeva et al. (2018) [31]	Kazakhstan	Policy Review	National accreditation system	Describes the development of Kazakhstan’s unique, ISQua-recognized national accreditation system.
Tsigengagel et al. (2021) [32]	Kazakhstan	Epidemiological Study	Baseline risk assessment	Provides epidemiological data on health-related offences, underscoring the need for robust safety frameworks like accreditation.

**Table 3:** Characteristics of Included Publications (*N* = 80).

Publication Type	Quantity	Percent (%)
Original research (observational)	29	36.25%
Systematic reviews	3	3.75%
Comparative studies	15	18.75%
Description of implementation experience	18	22.50%
Official documents and manuals	4	5.00%
Analytical reviews	9	11.25%
Qualitative studies	2	2.50%
Total	80	100.0%

Some publications relate to several thematic areas.

**Table 4:** Distribution of publications by subject area.

Thematic Area	Number of Publications
The Impact of Accreditation on Clinical Outcomes	18
Impact on organizational processes and security	15
Impact on patient satisfaction	11
Impact on medical personal	11
Economic aspects of accreditation	5
Experience of implementing JCI in various countries	11
Hospital-substituting technologies in surgery	15
Specific clinical areas (oncology, ophthalmology, etc.)	9
Systematic reviews and meta-analyses	4
Total mentions	99 *

Note: * The total number of mentions exceeds the total number of unique publications (N = 80) because individual sources were mapped to multiple thematic areas (e.g., studies addressing both clinical outcomes and patient satisfaction simultaneously).

**Table 5:** A Proposed Evaluation Framework for JCI-Accredited OSCs.

Domain	Specific Measure	Target	Data Collection Frequency
Patient Safety	Surgical site marking compliance	100% *	Monthly audit
Infection Control	Hand hygiene compliance	>90%	Quarterly observation
Clinical Outcomes	Unplanned hospital transfer rate	<2%	Monthly tracking
Patient Experience	Patient satisfaction (overall)	>4.5/5	Post-discharge survey
Efficiency	Procedure time (standardized cases)	Track trend	Quarterly analysis

* Note: Target reflects the patient-safety requirements concerning correct-site, correct-procedure, and correct-patient surgery under IPSG 4.

## Data Availability

No new data were created or analyzed in this study. Data sharing is not applicable to this article.

## Supplementary Materials

The following supporting information can be downloaded at: https://www.mdpi.com/article/10.3390/healthcare14172858/s1. Table S1. Complete Search Strategies for All Consulted Databases (January 2000–December 2025); Table S2. Comprehensive Data Charting and Thematic Synthesis of All 80 Included Publications.

## Abbreviations

AACI	American Accreditation Commission International
ACHSI	Australian Council on Healthcare Standards International
CIS	Commonwealth of Independent States
HRT	hospital-replacing technology
IEEA	International Society for Quality Assessment in Healthcare
ISQua	International Society for Quality in Health Care
JCAHO	American Joint Commission on Accreditation of Healthcare Organizations
JCI	Joint Commission International
OJSC	Open Joint-Stock Company
OSC	outpatient surgery center
RCHD	Republican Center for Healthcare Development
