# Supplementary material for: JCI Accreditation and Outpatient Surgery: A Scoping Review of Evidence on Healthcare Quality, Risk Management, and Policy
Source: Healthcare (Basel). 2026 Sep 4;14(17):2858. doi: 10.3390/healthcare14172858 (PMC13565535; doi:10.3390/healthcare14172858)
Supplement: Supplementary file 1 [file healthcare-14-02858-s001.zip › healthcare-4398768-supplementary.pdf]

Supplementary Materials

JCI Accreditation and Outpatient Surgery: A Scoping Review of Evidence on Healthcare Quality, Risk Management, and Policy

Kaisar Kudabayev, Aigul Ismailova, Kenesh Dzhusupov, Bakhyt Yeleussizova, Gulmira Zhauarova and Oxana Tsigengagel

Table S1. Complete Search Strategies for All Consulted Databases (January 2000–December 2025).

Database	Search Strategy / Full Search String	Filters / Limits	Records
**PubMed / MEDLINE**	(("Joint Commission International" OR "JCI") AND ("ambulatory surgery" OR "outpatient surgery" OR "day surgery" OR "one-day surgery" OR "ambulatory surgical center" OR "outpatient surgical center" OR "OSC" OR "office-based surgery") AND ("patient safety" OR "risk management" OR "healthcare quality" OR "clinical outcomes" OR "accreditation impact"))	• Language: English, Russian<br>• Date: 2000/01/01–2025/12/31	384
**Scopus**	(TITLE-ABS-KEY("Joint Commission International" OR "JCI") AND TITLE-ABS-KEY("ambulatory surgery" OR "outpatient surgery" OR "day surgery" OR "ambulatory surgical center" OR "outpatient surgical center") AND TITLE-ABS-KEY("patient safety" OR "risk management" OR "healthcare quality"))	• Language: English, Russian<br>• Year: 2000–2025	412
**Web of Science (Core Collection)**	TS=(("Joint Commission International" OR "JCI") AND ("ambulatory surgery" OR "outpatient surgery" OR "day surgery") AND ("patient safety" OR "risk management" OR "healthcare quality"))	• Language: English, Russian<br>• Timespan: 2000–2025	321
**eLIBRARY.ru (RSCI)**	("аккредитация JCI" OR "Объединенная международная комиссия" OR "JCI") AND ("амбулаторная хирургия" OR "дневной стационар" OR "однодневная хирургия" OR "стационарозамещающие технологии")	• Years: 2000–2025.	145
**Total**			**1262**

Table S2. Comprehensive Data Charting and Thematic Synthesis of All 80 Included Publications.

No.	Author (Year) [Ref]	Country/Region	Study Design	Evidence Stream / Focus Area	Key Findings & Risk Management Insights
1	Kasyanova (2022) [1]	Russia	Narrative Review	JCI Implementation	Analyzes the organizational possibilities of adapting JCI safety goals into Russian clinical management.
2	Kondratova (2015) [2]	Russia	Methodological Review	Patient Safety Goals	Investigates compliance with JCI International Patient Safety Goals (IPSG) for clinical risk mitigation.
3	Khairullin (2021) [3]	CIS / Global	Comparative Review	JCI in CIS clinics	Provides an overview of JCI implementation in CIS clinics, highlighting organizational and financial barriers.
4	Joint Commission International (2021) [4]	USA	Official Website / Manual	Accreditation guidelines	Details the standard operational requirements and safety frameworks for JCI healthcare accreditation.
5	Shilnikova (2014) [6]	Russia	Evaluative Study	Outpatient surgery center	Evaluates clinical and economic effectiveness of an outpatient surgery center as a hospital-replacing model of care.
6	Lobkarev (2017) [7]	Russia	Retrospective Analysis	Day hospital efficiency	Documents the operational and bed-occupancy efficiency in day surgical departments of multidisciplinary hospitals.
7	Mekshina (2024) [8]	Russia	Clinical Study	Outpatient surgery	Examines outpatient surgery treatments and patient care protocols in regional clinical hospitals.
8	Yarikov (2024) [9]	Russia	Narrative Review	Outpatient surgery	Highlights modern capabilities, efficiency, and clinical advantages of outpatient surgery centers.
9	Tsigengagel (2021) [32]	Kazakhstan	Epidemiological Study	Offences against health	Examines regional epidemiology of health-related offences to establish systemic risk assessment baselines.
10	Arksey (2005) [13]	UK	Methodological	Scoping review framework	Proposes the foundational five-stage framework for conducting scoping reviews.
11	Tricco (2018) [14]	Canada	Methodological	PRISMA-ScR guidelines	Outlines the PRISMA extension for scoping reviews checklist and reporting requirements.
12	ISQua (2021) [16]	Ireland	Official Website / Manual	External evaluation	Provides accreditation standards and peer-review resources for international quality evaluation.

13	Donahue (2000) [11]	USA	Conceptual Review	JCI evaluation models	Compares JCI accreditation with other international quality and safety evaluation frameworks.
14	Accreditation Canada (2021) [17]	Canada	Official Website / Manual	Accreditation resources	Outlines Canadian healthcare quality improvement and clinical risk assessment standards.
15	ACHS International (2021) [18]	Australia	Official Website / Manual	Quality evaluation	Provides Australian healthcare quality standards and patient safety benchmarking guidelines.
16	AACI (2021) [19]	USA	Official Website / Manual	Quality standards	Details American accreditation standards for patient safety and clinical quality management.
17	Chun (2020) [21]	South Korea	Pre-post Comparison	In-hospital mortality	Finds that JCI accreditation is associated with lower mortality in major cardiovascular disease patients.
18	Lam (2018) [20]	USA	Observational Study	Patient outcomes	Demonstrates a significant association between hospital accreditation and reduced 30-day mortality.
19	Kato (2021) [33]	USA	Comparative Study	Accrediting agencies	Compares quality outcomes across different accrediting bodies, showing small differences in high-baseline systems.
20	Ko (2020) [34]	Taiwan	Retrospective Study	ACS outcomes	Demonstrates that national accreditation is associated with improved clinical outcomes in acute coronary syndrome.
21	Hussein (2021) [35]	Global	Systematic Review	Accreditation quality	Finds that accreditation is generally associated with improved clinical, organizational, and safety outcomes.
22	Alhawajreh (2023) [22]	Global	Systematic Review	Quality improvement	Confirms positive, though context-dependent, impacts of accreditation on standard quality improvement indicators.
23	Campra (2021) [65]	Italy	Observational Study	Patient outcomes	Finds no significant differences in mortality between JCI-accredited and non-accredited hospitals in Italy.
24	Brubakk (2015) [80]	Norway	Systematic Review	Complex interventions	Outlines the challenges of measuring the impact of complex quality improvement interventions like accreditation.
25	Vuohijoki (2025) [76]	Finland	Systematic Review	Occupational health	Finds that JCI accreditation improves occupational health, safety culture, and patient safety indicators.
26	Bergholt (2021) [36]	Denmark	Cohort Study	Patient support	Shows that patients experience higher support and participation levels after first-time hospital accreditation.

27	Sperling (2020) [37]	Israel	Qualitative Study	Patient rights	Discusses how hospital accreditation standards promote and institutionalize patient rights.
28	Al-Sayedahmed (2021) [38]	Saudi Arabia	Benchmarking	Patient safety culture	Finds JCI accreditation associated with significant improvements in patient safety culture scores.
29	ElLithy (2023) [39]	Saudi Arabia	Retrospective	Medication incidents	Analyzes medication incident reporting trends in a JCI-accredited university teaching hospital.
30	Aliyeva (2025) [72]	Kazakhstan	Cross-Sectional	Satisfaction disparities	Documents disparities in obstetric satisfaction between insured and uninsured patients under OSMS.
31	Tarieh (2022) [46]	Jordan	Case Study	Staff productivity	Shows that JCI accreditation is associated with improved staff productivity and clinical motivation.
32	Aboshaiqah (2016) [41]	Saudi Arabia	Comparative Study	Quality of care	Compares patient satisfaction and perceived quality of care in hospitals with and without accreditation.
33	Dasan (2023) [42]	Palestine	Master's Thesis	Patient satisfaction	Evaluates the effect of JCI accreditation implementation on perceived patient satisfaction levels.
34	Barghouthi (2018) [40]	Palestine	Comparative Study	Patient satisfaction	Finds significantly higher patient satisfaction in JCI-accredited Palestinian hospitals compared to non-accredited ones.
35	Aliyeva (2025) [73]	Kazakhstan	Qualitative Study	Obstetrics challenges	Investigates healthcare professionals' perspectives on obstetric-gynecological care challenges in Kazakhstan.
36	Kwan (2021) [43]	South Korea	Cross-Sectional	Safety culture	Explores nurse perceptions of patient safety culture and their correlation with JCI accreditation.
37	Despotou (2019) [44]	South Korea	Quantitative Study	Safety culture	Evaluates nurses' perceptions of JCI accreditation on patient safety practices in South Korean hospitals.
38	Elkins (2010) [45]	USA	Survey	Staff stress	Analyzes perceived stress and workload among nursing staff during the hospital accreditation process.
39	Aliyeva (2025) [77]	Kazakhstan	Multivariate Analysis	Satisfaction predictors	Identifies key clinical communication and accessibility predictors of patient satisfaction in Almaty gynecological clinics.
40	Zhang (2024) [47]	China	Cross-Sectional	Safety culture	Examines hospital employees' perceptions of JCI accreditation and safety culture before and after re-accreditation.

41	Zhang (2023) [23]	China	Qualitative Study	Leader perceptions	Identifies brand reputation, clinical safety, and organizational branding as key motivators for Chinese hospital leaders.
42	Algahtani (2017) [48]	Saudi Arabia	Survey	Perception of JCI	Investigates healthcare professionals' perceptions of JCI accreditation impact on quality.
43	Hamed (2023) [49]	Palestine	Master's Thesis	Nurse satisfaction	Compares job satisfaction and patient safety culture between JCI and non-accredited Palestinian hospitals.
44	Rhoden (2021) [24]	Brazil	Survey	Nurse stress	Evaluates musculoskeletal pain and stress among Brazilian nurses during the preparation phase for JCI.
45	Kakemam (2020) [50]	Iran	Survey	Staff attitudes	Examines Iranian hospital employees' attitudes toward accreditation and safety culture.
46	Agustine (2019) [51]	Indonesia	Review	Quality perception	Synthesizes Asian health professionals' perceptions of JCI impact on clinical outcomes.
47	Sriratanaban (2020) [27]	Thailand	Economic Study	Economic aspects	Details implementation costs, structural demands, and mixed financial returns of JCI in Thailand.
48	Day (2013) [28]	Guatemala	Case Study	Limited resources	Proves JCI standards can be successfully adapted to improve pediatric oncology care in resource-limited settings.
49	Kobayashi (2021) [53]	Japan	Retrospective	Orthopedic surgeons	Examines Japanese surgeon compliance with JCI surgical safety checklist and clinical protocols.
50	Milanesi (2025) [54]	Italy	Implementation Report	Oncology care	Standardizes cancer patient pathways using JCI and ISO guidelines for clinical safety.
51	Sallam (2023) [55]	UAE	Survey	Medication safety	Evaluates medication safety and reconciliation practices in Dubai's JCI-accredited pharmacies.
52	Ministry of Health (2009) [66]	Kazakhstan	Policy	National standards	Approves national accreditation standards for healthcare entities in the Republic of Kazakhstan.
53	President of Kazakhstan (2010) [67]	Kazakhstan	Decree	Salamatty Kazakhstan	Launches state program for quality and health system modernization.
54	Kaupbaeva (2018) [31]	Kazakhstan	Policy Review	Accreditation system	Reviews the evolution of Kazakhstan's national healthcare accreditation system.

55	Kulkaeva (2025) [82]	Kazakhstan	Policy Review	Barriers	Analyzes barriers and strategic opportunities for national accreditation development.
56	ACQH (2021) [68]	Kazakhstan	Website	Quality center	Details resources and standards provided by the National Accreditation Center for Quality in Healthcare.
57	Aiypkhanova (2021) [69]	Kazakhstan	Policy Review	Independent quality	Explores transferring national accreditation to an independent competitive environment.
58	Ermukhanova (2015) [56]	Kazakhstan	Review	Hospital-replacing tech	Outlines regional development and medical necessity of day-hospital/ambulatory technology.
59	Belevitin (2016) [57]	Russia	Review	Outpatient surgery	Discusses modern clinical capabilities and surgical indications in day hospitals.
60	Karailanov (2016) [58]	Russia	Evaluation	Day hospital beds	Proposes a rational use framework for day surgical beds in multidisciplinary clinical settings.
61	WHO (2018) [15]	Global	Policy report	Quality directive	Outlines a global imperative for high-quality, safe, and patient-centered service delivery.
62	Devkaran (2014) [64]	UAE	Interrupted Time Series	Clinical documentation	Proves JCI accreditation is associated with improved clinical documentation compliance.
63	Vorobyov (2024) [59]	Russia	Review	Surgical tech	Evaluates technological developments in outpatient surgeries to optimize inpatient clinical resources.
64	Aituganova (2017) [60]	Kazakhstan	Evaluation	Outpatient quality	Assesses service quality and clinical protocols in day surgical departments of regional medical centers.
65	Auelbekova (2023) [70]	Kazakhstan	Evaluation	Surgical quality	Evaluates patient safety indicators and clinical outcomes in ambulatory surgery departments.
66	RCRD (2021) [71]	Kazakhstan	Website	Accreditation	Details resources, guidelines, and standards of the Republican Center for Healthcare Development.
67	Inomata (2018) [74]	Japan	Retrospective	Operating room times	Evaluates JCI accreditation's impact on operating room time management.
68	Okumura (2019) [29]	Japan	Observational	Cataract surgeries	Shows that standardization via JCI significantly shortens cataract surgery times.

69	Myler (2025) [61]	USA	Clinical Review	Ambulatory outcomes	Synthesizes current clinical outcomes and safety profiles in ambulatory surgical procedures.
70	Fleisher (2023) [30]	USA	Economic Review	Ambulatory economics	Analyzes cost-saving potential and policy models of perioperative ambulatory care.
71	Al-Awa (2012) [25]	Saudi Arabia	Benchmarking	Patient safety culture	Benchmarks safety culture post-accreditation, finding higher scores in JCI hospitals.
72	Gridnev (2021) [62]	Russia	Process model	Outpatient surgery	Proposes a new operational process model for outpatient surgery departments to increase clinical efficiency.
73	Hwang (2021) [12]	South Korea	Case report	Small-scale clinic	Documents JCI accreditation implementation in a small outpatient clinic.
74	Falstie-Jensen (2022) [5]	Denmark	Population study	Accreditation cycles	Analyzes the quality of care across consecutive cycles of accreditation.
75	Bharara (2020) [75]	India	Evaluation	Hand hygiene	Evaluates hand hygiene compliance in an ICU preparing for accreditation.
76	Greenfield (2019) [26]	Australia	Multicenter Study	Clinical care change	Investigates how accreditation stimulates local organizational improvements.
77	Hashemi (2014) [78]	Iran	Audit	Emergency department	Evaluates emergency department accreditation based on JCI standards.
78	Song (2014) [52]	China	Interrupted Time Series	Stewardship	Shows positive impact of JCI on outpatient antibacterial stewardship.
79	Shaw (2003) [10]	UK	Review	Evaluating accreditation	Discusses the theoretical basis and models of evaluation in healthcare accreditation systems globally.
80	Lebedev (2017) [63]	Russia	Evaluation	Day surgery	Evaluates hospital-replacing technologies in a clinical diagnostic center.
